# Collectanea

**Published:** 1832-02

**Authors:** 


### COLLECTANEA.

Floriferis ut apes in saltibus omnia libant,

Omnia nos, itidem, depascimur aurea dicta.

### PATHOLOGY.

*Cases in Surgery.* By David L. Rogers, m.d. of New-York.

Case I. *Abdominal Abscess.* I was requested, in July, 1830, to visit Mr. Jarvis, a ship-carpenter, aged about forty-five years, in connexion with Doctors Baldwin and Rockwell. Mr. Jarvis was a man of temperate habits and of robust constitution. He had for several years been troubled with a reducible inguinal hernia on the left side, for which he had worn a truss. He had been seriously indisposed for several days previous to my seeing him, with what was supposed a constipation of the bowels, and the means usually employed for the relief of such complaints had been most actively employed by the gentlemen in attendance. I visited him on the 1st day of July, when he presented the following symptoms: countenance indicative of great irritation, pulse full and soft, tongue covered with a dark brown sordes, the skin moist, with a warm, clammy perspiration, the bowels much tumefied, and painful on the slightest pressure, particularly in the right inguinal region, which was the original seat of pain, urine high coloured and small in quantity, the bowels torpid, and difficult to be acted upon by the most drastic cathartics, although he would occasionally have a small discharge, partaking of the properties of the medicines. The stomach was generally retentive, but was sometimes disposed to reject its contents. The hernia on the left side was soft and natural, and easily returned, and could not be considered as the cause of irritation. This case, from its obscure character, excited much interest; its strong resemblance to strangulated hernia, connected with the fact of his being predisposed to that disease, rendered the diagnosis extremely difficult, as some of the most prominent symptoms of strangulated hernia were wanting. The condition of the stomach, the pulse and fæcal discharges, offered strong arguments against this opinion.

On closely examining the right inguinal region, at the part where he complained of the greatest pain, a small tumor could be discovered about the size of a filbert, in the immediate situation of the internal ring; but increasing the pressure, it immediately disappeared. As no definite idea could be formed as to the nature of the disease, the symptoms being violent, and threatening to prove fatal, unless some relief could be afforded, I proposed cutting cautiously down to the internal ring, to ascertain the character of the tumor, supposing that it might possibly be a section of intestine strangulated, and yet the intestine remain sufficiently pervious to give passage to the fæces. It was, however, concluded to postpone the operation until the next day, and to repeat the treatment pursued by the attending physicians, as bleeding, warm bath, calomel, enemas, and hot fomentations to the bowels.

July 2d. No relief has been obtained by the treatment adopted yesterday: some of the symptoms are much aggravated; he had no sleep, with much more sickness at stomach; tongue black; considerable delirium during the night. The critical situation of the patient admitted of no delay, although the symptoms of hernia were very obscure, and it was doubtful if any existed; yet in his situation almost any practice would be proper, that could offer the most distant prospects of preserving life. With this understanding, I commenced the operation, and performed it in the usual mode of operating for hernias at the internal ring, in the presence of Doctors Mott, Baldwin, and Rockwell; the details of which it is unnecessary to mention. On arriving at the internal ring, we found a protrusion of peritoneum, in all respects resembling a hernial sac. I accordingly proceeded to open it, with all the caution observed in such cases, when, to the astonishment of all present, the moment an opening was made, a volume of most fetid matter rushed out with a force that projected it two or three feet; more than a quart was immediately discharged. The mystery of the case was explained; the tumefaction of the abdomen subsided in a few days; the tongue cleared off; the bowels became active and regular, and in a month the discharge ceased; the wound healed, and he returned to his usual occupation in good health.

CASE II. *Abdominal Abscess.* The following claims much interest, as it has some relation to Case 1st, although proceeding from a different cause, and the symptoms being more determinate of its existence. This case came under the care of my friend Dr. M'Caffry, who has obligingly furnished me with its history, which I shall give in his own words.

"Mrs. S., lately from England, residing at 282, Division street, was, after a tedious and difficult labour, delivered of her eighth child, late in the month of November, 1830. Her lochial discharges, which ceased much earlier than formerly, were succeeded by cough, pain and oppression in the chest, accompanied with fever; which rendered depletion both local and general necessary, which was but in part carried into practice. In the mean time she was seized with excruciating pain in the right inguinal region, extending through the iliac, sacral and lumbar regions, depriving her of the power of lying on that side, or using the right leg.

"Although there was no swelling, or any symptoms resembling phlegmasia dolens, evening exacerbations and night sweats succeeded the foregoing symptoms; no relief could be given to the sufferer, although attended by two or three respectable physicians for the space of twelve weeks. At this time I was requested to visit her. I found her weak and emaciated, and suffering from fever and sweats. I paid attention to the bowels, and put her upon the tonic treatment, but she obtained no relief. Upon examining the seat of the pain, I discovered a deep-seated tumor, and something resembling a fluctuation of matter. Not satisfied with my own views of the case, I requested the attendance of Dr. D. L. Rogers."

According to Dr. M'Caffry's request, I visited Mrs. S., and found her situation much as he has detailed it. The tumor in the side possessing strong symptoms of containing an abscess, added to the throbbing pain in the tumor, the indistinct fluctuation, and the general symptoms of hectic, I was well satisfied that Dr. M'Caffry was correct in his opinion, that a large abscess existed in the cavity of the peritoneum. It was proposed to evacuate the matter: to this the patient readily acceded. She was laid on the side of the bed, partly on her left side. With a common scalpel, I made an incision in the direction of the posterior spine of the ilium, between two and three inches in length, dividing the external oblique, internal oblique, and transversalis muscles. On dividing the last-named muscle and its fascia, the peritoneum immediately protruded through the opening. With a pair of forceps I cautiously made an opening through it, when a pint of matter immediately passed out. Not having visited the patient after the operation, Dr. M'Caffry concludes his report thus:

"In about five days the discharge entirely ceased, and the wound closed up; her fever and sweats disappeared, her appetite returned, and in two weeks from the discharge of the pus, she was able to pursue her domestic concerns, although she had been fifteen weeks confined to bed, on the day the abscess was opened."

*Remarks.* Having examined several females after death, who died of what Dr. M. Hall would term irritative fever, I have in all such cases found a large quantity of seropurulent effusion, mixed with flakes of coagulable lymph. In these cases, I have remarked the great abdominal distention by flatus. The sensibility of the intestines being destroyed by the morbid action which is going on around them, it thus happens that it requires the most stimulating cathartics to affect them. These remarks are intended to direct the attention of physicians to the subject. In many cases, by close observation, we might detect the existence of matter in some part of the abdomen, and, by timely discharge, the patient might be rescued from danger. Many practitioners hesitate in opening abscesses within the cavity of the abdomen, preferring to leave them to be discharged by the ulceration of the integuments, when in most cases the patient sinks under the irritation before the process is completed.

This delay proceeds from two causes, equally unfounded and absurd. First, it is believed by some, that wounds of the peritoneum are attended with peculiar danger; they prefer avoiding the responsibility, and to leave it to nature. Every day's practice teach us a different lesson; large hernial sacs are opened, and the intestines returned, tumors of a large size are removed from its cavity, and wounds are daily occurring in which this membrane is even lacerated, and yet these cases recover without being much incommoded by inflammation. Secondly, it has for a long time been the opinion of practitioners, that an abscess should have a certain degree of maturity before it is opened, which in common language is termed, bringing them to a head; others again believe, that abscesses will do best to be left to break of themselves, and to this rule they make but few exceptions. I have been in the habit, for several years, of discharging the matter as soon as I was satisfied of its presence. I believe there is no rule more injurious in practice, than the opinion that matter must be left to approach the surface, and excite a certain degree of inflammation there, before it would be proper to open the sac. The integuments being inflamed, the puncture must be much more painful than in the natural state, and having become thin by absorption, it is much more difficult to heal the wound; and in fact, the integuments having lost their support by the discharge of matter, the edges fall in, a slough follows, and the result is, you have to heal the wound by granulations from the bottom. This is observed in buboes, which are allowed to break, and in abscesses about the face and neck, leaving the most unpleasant scars; whereas, if abscesses are opened early, previous to the integuments being affected, the incision may be immediately healed, by bringing the edges in contact, and the abscess cured by bringing its sides in coaptation, by well adjusted compresses; or, should it be necessary to keep the wound open for a few days, no injury will happen to the integuments; the wound will not enlarge, and when the necessity ceases for keeping the wound open, it may be closed, and a simple line will mark its situation.

In some cases, the lives of persons are hazarded by this delay in opening abscesses, as when matter is situated under the tendon of the occipito-frontalis muscle, or the fascia of the leg, arm, and theca of the fingers. In 1829, I examined a man who had a severe attack of erysipelas of the leg; he had recovered from the inflammation, but died of some irritation which was not understood. The head, chest, and abdomen appeared in a sound state; a large abscess was found under the fascia of the leg, which was no doubt the cause of death. But notwithstanding the diversity of opinions upon the subject of abscesses in general, we have but one rule in relation to those within the cavity of the peritoneum, which is, in all cases, to cut boldly down and discharge the matter, the moment we are satisfied of its presence.—*New-York Med. Journal.*

### *Microscopical Observations on the Blood in its Pathological State.*

When the doctrines of the humoral pathology were discarded, and tenets of a very opposite description took possession of the schools, the state of the blood and other fluids became most culpably neglected; and in throwing aside the errors of the obsolete pathology, it is exceedingly probable that we, at the same time, too hastily surrendered its truths. Such appears, now at least, to be the opinion; and the blood is again, and properly, rising into an importance in pathology which it has not for a considerable time enjoyed. The chemical examination of this fluid, in typhoid and other fevers, has very distinctly proved that its composition is altered in these diseases, and has, in the eyes of some, been the means of indicating their proper treatment; and the extended observation of M. Andral has furnished him with proofs of a morbid state of the blood being the cause of many diseases and modifications of disease, and led him, in his Pathological Anatomy, to make it the subject of special consideration. The paper, however, we are about to refer to, does not make the chemical composition of the blood the test of its diseases, but has recourse to the somewhat novel idea (so far as relates to pathology) of submitting it to microscopical examination, and thus ascertaining the changes which its particles undergo in different affections. The results at which the author of this paper, M. Donné, has arrived are interesting in more than one point of view, and we shall, on that account, lay the following brief abstract of them before our readers.

It is well known that the blood is composed of globules swimming in a colourless and transparent fluid or serum. The shape of these globules varies in different animals; in man they are nearly spherical, and this form is constant. But the contour of these globules, so defined and distinct in the healthy subject, becomes (according to the experience of M. Donné,) generally altered and deformed in the course of most diseases. In an individual in good health, the globules are perfectly rounded, circumscribed by an obscure line, which is removed when they are placed on a plate of glass, and present a transparency in the centre, while their diameters are sensibly equal. On the other hand, in a subject exhausted by long suffering, and whose organs have undergone decided disorganization, the sanguineous globules are less numerous, are smaller and ill-shaped, have their outline flayed and ill-defined, and this equality and regularity in their diameters is no longer to be observed. In illustration of these points, M. Donné cites the following examples:

"1st. Blood of a female, æt. twenty-six; death from gangrene of the lung: globules of the blood small and ill-shapen, their outline ragged.

"2d. Blood of a female who died from puerperal metro-peritonitis, (an extensive application of leeches having been made:) on dissection, a small quantity of blood only was found; there was sero-purulent effusion into the abdomen, and great softness of the liver and all the other organs; the globules were less deformed than in the preceding case, but were very ill defined; the liquid effused in the abdomen presented some globules scattered through it, of very irregular shape.

"3d. Serum of the blood of a female who had long laboured under disease of the brain, and was bled for erysipelas: globules very small and rare.

"4th. Blood of a man bled for bilious fever and pneumonia: globules beautiful, tending to run together and agglutinate.

"5th. Blood of a female who died dropsical: globules extremely small in number.

"6th. Serum of the blood of a young girl labouring under bilious fever: globules well marked, but little transparent.

"7th. Blood of a female cut off by disease of the liver: globules pretty natural, but beginning to become deformed.

"8th. Blood of a young man, dead from acute peritonitis, treated by mercurial frictions: globules very deformed.

"Blood of a young man cut off by the same disease, treated in a similar manner: natural form of the globules entirely lost, some of them very large."

Such are a few of the facts adduced by M. Donné, and though he merely contents himself with their announcement, without drawing any conclusions from them, we may be permitted to remark that, along with the collateral evidences which are daily submitted to us, they afford strong proofs of the blood itself being subject to diseased changes, and offer great encouragement to extend research in this direction.—*Glasgow Med. Examiner.*

---

*Enormous Dilatation of the Biliary Ducts.* M. Berard has met with a case in which the biliary ducts were enlarged from twelve to fifteen times their natural size in the parenchyma of the liver. The patient had neither icterus nor obliteration of the ductus choledochus, but the biliary ducts contained many calculi.—*Rev. Méd.*

---

### MATERIA MEDICA.

*Combination of Nitre and Calomel.* M. Burdach states, in a recent German Journal, that the addition of nitrate of potash prevents calomel from producing salivation, the nitre causing its prompt expulsion by stool. This combination he also asserts to be a powerful derivative, and relieves the head, the chest, and the liver, more effectually than either of them will do separately. Certain diseases, as hydrocephalus, croup, &c. he adds, require large doses of calomel, and if this medicament is not eliminated from the system, it becomes a poison: the addition of nitre prevents this unfortunate result.—*Gazette Médicale.*

---

*Corrector of Opium.* According to M. Puchelt, a German physician, the sulphate of soda is an excellent corrector of the unpleasant effects of opium, given in the proportion of a scruple to half a grain of opium. This dose may be repeated two or three times a day. In combination with Glauber's salt, opium, he says, may be administered in cases where slight plethora, local or general, prevents recourse being had to opium alone; in obstinate hemorrhages, principally, this mixture will produce the happiest effects. But if sulphate of soda prevents the congestion which opium sometimes produces, M. P. says that there is another article which corrects its narcotic, without diminishing its sedative effects; this is the castor. The combination of opium and castor he considers very useful in cases of hysteria.—*Ibid.*

---

### PRACTICAL MEDICINE.

*Endermic Application of Morphia in Cases of Rheumatism of the Joints* Messrs. Trousseau and Bonnet have recently published in the Archives (Nov. 1831,) many cases of rheumatism of the joints, which were successfully treated in this manner. For the purpose of detaching the epidermis, they recommend a pommade composed of equal parts of concentrated ammonia and lard, and one fifth part of suet. This application is preferred to cantharides, on account of the rapidity with which it acts, and the facility with which its effects are limited. The acetate, hydrochlorate, and sulphate of morphia were used; but the two latter were particularly selected, for their greater solubility, and consequently more ready absorption. The spot chosen for the application of the remedy should be as near the seat of pain as possible. The quantity of morphia applied should be from one to two grains, according to the effects produced.

### SURGERY.

*Diagnosis of Hydrocele.* M. Segalas recently submitted to the Académie de Médecine a new method of ascertaining the transparency of the parts in doubtful cases of hydrocele: he applies to the scrotum one extremity of the eye tube which is used for exploring the urethra, and puts his eye to the other, and he thus detects the existence of fluid, if it be present. The result is explained by the isolated condition of the eye, the perpendicularity of the rays of light transmitted through the tube, and the pressure of the instrument against the scrotum. M. Roux doubts the utility of this method in certain cases, as when the tunica vaginalis is much thickened.—*Gaz. des Hôpitaux.*

The Editor of the London Medical Gazette observes, and we think correctly, "that M. Roux's objection proves nothing; and perhaps the only circumstance that would tend to depreciate the utility of the new method is, that it enables us to see *too well*,—to discover transparency where such a thing could be least expected. With the tube and a candle, light may be perceived through the palm of the hand."

---

### MEDICAL JURISPRUDENCE.

*Poisoning with a Tobacco Clyster. (Hufeland's Journal der Praktischen Heilkunde.)* An interesting case of poisoning with a tobacco clyster is related by Dr. Grahl, of Hamburgh, in the Journal here quoted. The individual, a female twenty-four years of age, was liable to dyspeptic symptoms and obstinate constipation, on account of which she had been for a few days under the care of the relater, and with considerable advantage. One day the patient's mother proposed that a tobacco clyster should be administered, which Dr. Grahl peremptorily prohibited. Nevertheless, on the following day she took the advice of a female quack of her acquaintance, who recommended a clyster made with an ounce or an ounce and a half of tobacco, boiled for fifteen minutes with a sufficient quantity of water. In two minutes after it was administered, the patient was seized with vomiting, violent convulsions, and stertorous breathing, which gradually became weaker and weaker till she died, three quarters of an hour after the clyster was administered. The following were the appearances remarked in the body, which was examined two days after death: Great lividity of the back, slight lividity of the abdomen, retraction of its anterior parietes, paleness of the lips, firm closure of the jaws, flexibility of the joints; the omentum very red, without gorging of its veins; the small and great intestines, both outside and inside, gorged with blood, and red; and in some parts of the mucous membrane extravasated bloody patches; the great vessels of the abdomen more empty of blood than usual: the stomach natural: the lungs pale red: the heart empty of blood, in both sides: the brain quite natural, and without any accumulation of serosity in the ventricles. —*Edinburgh Med. and Surg. Journal.*

---

### MIDWIFERY.

*Cæsarean Operation successfully performed. (Archives Générales de Médecine.)* An extract is given in this Journal from a late thesis of a Parisian graduate, M. Jolly, giving an account of the remarkable success which his father, a surgeon at Chateau-Thierry, has experienced in performing the Cæsarean operation. He has operated six times, five of his patients being country women, and the sixth an inhabitant of the town. In all, the labour had lasted at least forty-eight hours before the operation was performed, and the waters had been discharged. In one patient only of the six no fatiguing attempts had been made by midwives or accoucheurs to finish the labour. He always made the incision on the linea alba, between the navel and pubes, and divided the uterus in the same direction, taking care to restore it first to the perpendicular position, if it was inclined. There was never any material hemorrhage; no patient, indeed, lost more than two ounces of blood. In dressing the wound he always had recourse to the gastroraphy, which, instead of producing the ill consequences usually ascribed to it, appeared to him always to contribute greatly to the cicatrization of the wound. In two of the six cases no untoward symptom whatever followed the operation, and the cure was perfected before a month expired; in two others a smart degree of inflammation of the abdomen supervened, but was successfully combated by venesection, baths, and fomentations; and the remaining two died evidently of metroperitonitis, one on the fourth day, the other at a later period, when there appeared every chance of her recovering under the antiphlogistic treatment. Of the six infants, four were born alive and survived, but two were dead after the operation was concluded, although they were thought to have been alive before it was performed. In no instance did hernia ensue; but there was always some prominence of the abdomen at the cicatrix, which had diminished from six inches in length to three only. This is the most favorable statement which has ever appeared on the subject of the Cæsarean operation.—*Ibid.*

