# Erratum

**Published:** 1832-03

**Authors:** 


Erratum *in p.* 169: for "Mophia," read "Morphia."

